# Deciphering Nano–Bio Interactions of Hyaluronic Acid-Clove Carbon Dots for Multifunctional Endodontic Nanotherapy

**DOI:** 10.3390/dj14070449

**Published:** 2026-07-17

**Authors:** Mohanprasanth Aruchamy, Natesan Thirumalaivasan

**Affiliations:** Applied Nano-Bio Therapeutics Laboratory, Department of Periodontics, Saveetha Dental College and Hospitals, Saveetha Institute of Medical and Technical Sciences (SIMATS), Saveetha University, Chennai 600077, Tamil Nadu, India; mohanbiochemist03@gmail.com

**Keywords:** dental Carriers, *Streptococcus mutans*, carbon dots, antibacterial, antibiofilm and biocompatibility

## Abstract

**Background:** *Streptococcus mutans (S. mutans)* plays a major role in dental biofilm-related infections and contributes to antimicrobial resistance. Therefore, the development of biocompatible nanomaterials with antibacterial, antibiofilm, and regenerative properties is important for dental applications. **Methods:** In this study, hyaluronic acid and clove extract were used as natural precursors to synthesize hyaluronic acid-clove carbon dots (HCCDs) through a hydrothermal method. The synthesized HCCDs were characterized by XRD, FTIR, TEM, SEM, SAED and EDAX analysis. The antibacterial and antibiofilm activities against *S. mutans* were tested. Cell viability tests, AO/PI staining, morphological observation and wound-healing assays were used to evaluate cytocompatibility in MG-63 cells. **Results:** A broad peak (23.449) observed from XRD coincided with an amorphous graphitic carbon structure. TEM images showed the presence of a spherical nanostructure with an average size of 4 ± 2 nm. The FTIR result verified the presence of hydroxyl, carbonyl and oxygen-containing functional groups on the HCCD surface. Their synthesized HCCDs exhibited noteworthy antibacterial and antibiofilm activity with an MIC of 62.5 µg/mL against the *S. mutans*. Low toxicity toward MG-63 cells was observed, with 86% cell viability at 200 µg/mL in the cytocompatibility study. Additional good cellular compatibility was confirmed using AO/PI staining and morphological analysis. Furthermore, normal cell migration was observed, as wound healing assays showed no significant difference in closure between the treated and control groups. **Conclusion:** HCCDs exhibited antibacterial, antibiofilm, biocompatible, and wound-healing properties, highlighting their potential for dental nanomedicine and the treatment of oral biofilm-associated infections.

## 1. Introduction

Dental diseases continue to be one of the most common chronic oral health issues in the world, especially dental caries and periodontal infections [1]. Bacterial biofilm formation is one of the main characteristics of dental caries, and microorganisms like *S. mutans* are important in acid production, formation of enamel demineralization and progression of oral infection [2]. In addition, the Biofilm on the tooth surface is an organized and dynamic process with significant implications for the development of dental caries and periodontal disease [3]. The acquired pellicle is a thin layer of salivary proteins and glycoproteins that initially covers the enamel surface and provides a substrate for the attachment of oral microorganisms. Bacterial colonization of the tooth surface initiates when bacteria begin to metabolise dietary sugars and produce extracellular EPS (mainly glucans), which bind to the tooth surface, particularly by early colonizers such as *S. mutans* [4]. These EPS components help to hold bacteria in place and facilitate the integration of other microbial communities, resulting in the formation of mature dental biofilms or dental plaque. The biofilm matrix confers protection on the bacteria, enhancing their survival, resistance to antimicrobials, resistance to host immune responses, and resistance to environmental stressors. The bacteria in the biofilm communicate via quorum sensing, and an acidic microenvironment in the biofilm leads to demineralization of enamel and the development of dental caries [5]. Additionally, biofilms can contribute to gingivitis, damage the gums, and result in chronic oral infections. Because of their complexity and resistance, dental biofilms have attracted researchers’ interest in developing new antibiofilm nanomaterials and therapeutic approaches to disrupt bacterial adhesion, EPS production, and biofilm maturation on tooth surfaces. Numerous antimicrobial agents and antibiotics are available to combat oral pathogens, but overuse can lead to antimicrobial resistance, changes in oral microflora, and side effects. In this context, dental research is paying great attention to the development of novel biocompatible nanomaterials with antimicrobial and antibiofilm properties [5,6].

Carbon dots (CDs) are a new type of zero-dimensional carbon nanostructures with a size mostly smaller than 10 nm, possessing special physicochemical and biological properties, such as excellent water solubility, tunable fluorescence, high photostability, ease of surface functionalization, high biocompatibility, etc. [7,8]. Based on these benefits, CDs have been extensively studied for biomedical applications such as bioimaging [9,10], biosensing [11], drug delivery [12], antimicrobial therapy [13], tissue engineering, and regenerative dentistry [14]. Because of their toxicity-free, eco-friendly, and biocompatible nature, green synthesis methods using natural biomaterials and plant-derived precursors have gained a growing interest in recent years. Naturally derived CDs can also contain many oxygen-containing surface functional groups, which can increase their interactions with biological systems and enhance their antimicrobial properties [15].

A polysaccharide, hyaluronic acid, is a naturally occurring polysaccharide that is present in the extracellular matrix and in the connective tissue. It possesses good biocompatibility, biodegradability, hydrophilicity and wound-healing properties that are beneficial in biomedical and dental applications [16]. Due to its ability to stimulate cell proliferation, remodel the extracellular matrix (ECM), and possess anti-inflammatory properties, HA has been previously used in periodontal therapy, oral wound healing, and periodontal tissue regeneration. The hydroxyl and carboxyl functional groups of hyaluronic acid are crucial in the creation of water-stable and cell-compatible functionalized carbon nanostructures during the hydrothermal synthesis process [17].

Clove (*Syzygium aromaticum*) is a medicinal plant that has gained popularity as a dental herb for its antimicrobial, analgesic, antioxidant, and anti-inflammatory properties [18]. The main compound in clove, eugenol, is used in numerous dental products; it has been used for many years as a temporary cement, a root canal cement, and a pain reliever [19]. Furthermore, clove extract contains a high concentrations of flavonoids, phenolic compounds, and tannins, which may serve as natural carbon sources and surface passivating agents for the synthesis of carbon dots [20]. Oral bacteria were found to be more sensitive, in terms of antimicrobial and antibiofilm activity, to plant-based carbon dots than other bacterial groups, an effect attributed to the synergistic activity of the phytochemical functional groups on their surface. The present study aims to synthesize and characterize HCCDs and to evaluate their antibacterial, antibiofilm, cytocompatibility, and wound healing potential for possible dental and biomedical applications.

## 2. Experimental

### 2.1. Materials and Chemicals

Hyaluronic Acid was purchased from Sigma-Aldrich, St. Louis, MO, USA. Mueller Hinton Agar [Catalogue No: M173, Brand: Himedia, Mumbai, India]. DMEM, Fetal Bovine Serum (FBS), Antibiotics, and Trypsin were procured from Gibco, Thermo Scientific, Waltham MA, USA, and all chemicals were purchased from a local authorized vendor.

### 2.2. Preparation of HCCDs

**Scheme 1:**
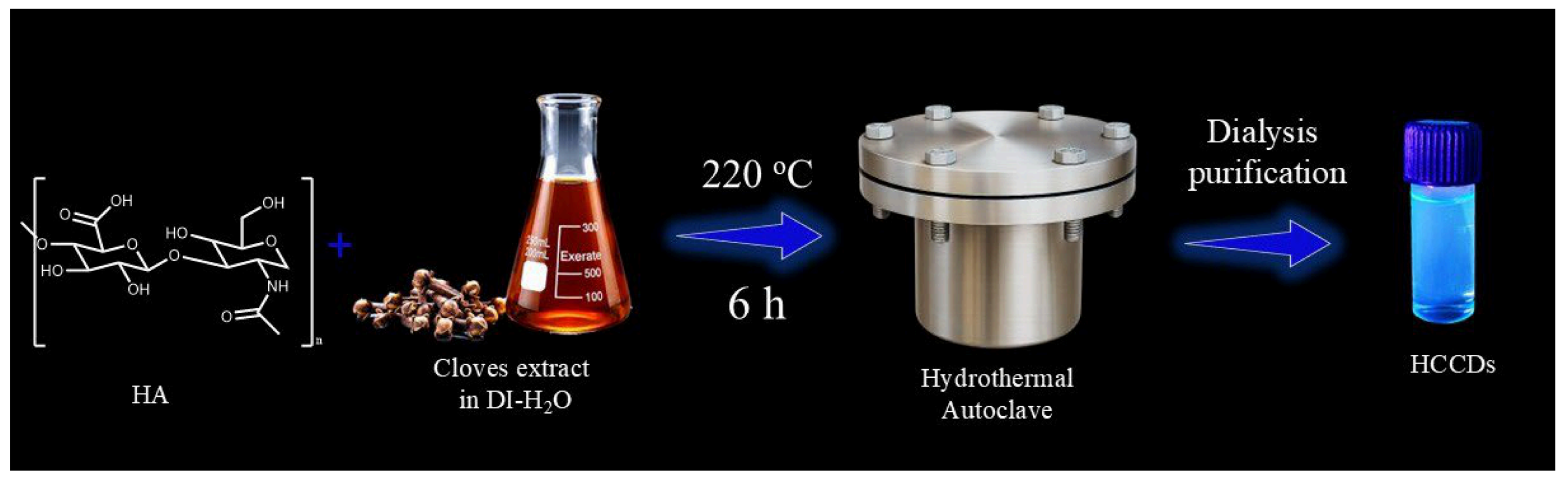
Schematic representation of the hydrothermal synthesis of HCCDs.

Clove extract was prepared by suspending 5 g of clove powder in 50 mL of deionized water (DI-H_2_O), followed by continuous agitation in an orbital shaker for 24 h to facilitate the extraction of water-soluble phytochemicals. After extraction, the mixture was filtered to remove insoluble plant residues, and the resulting aqueous clove extract was collected for further use. For the synthesis of HCCDs, 5 mL of the prepared clove extract was mixed with 200 mg of hyaluronic acid (HA) and dispersed in 20 mL of DI-H_2_O. The resulting mixture was thoroughly homogenized to obtain a uniform precursor solution, which was subsequently subjected to hydrothermal treatment for the synthesis of HCCDs. This mixture was then autoclaved at 220 °C for 6 h. The solution was brought down to room temperature, large particles were removed by centrifugation, and the resulting brown HCCD solution was dialyzed using a 1 KDa cutoff. The collected carbon dots were dried in a hot-air oven at 100 °C for 5 h (Scheme 1).

#### 2.2.1. Characterizations of HCCDs

Various analytical techniques were performed to characterize the synthesized Hyaluronic Acid and clove extract carbon dots, HCCDs, with respect to their physicochemical properties. UV–Visible spectrophotometric analysis (UV/Vis Lambda 365+, PerkinElmer, Waltham MA, USA) of the optical absorption properties was performed by dispersing the dried HCCD powder in distilled water, and the absorbance spectrum was recorded from 200 nm to 800 nm. The photoluminescence (PL) properties were investigated by a fluorescence spectrophotometer (FL 6500, PerkinElmer, USA) in which the emission spectra were recorded under different excitation wavelengths (200–450 nm) to study excitation-dependent fluorescence behavior. Fourier transform infrared spectroscopy (FTIR) analysis was performed using an FTIR Spectroscopy (Spectrum Two, PerkinElmer, USA) in the range of 4000–400 cm^−1^ to identify surface functional groups. The amorphous structure of the HCCDs was analysed using an X-ray diffractometer (D8 Advance, Bruker, Bremen, Germany) with Cu-Kα radiation (λ = 1.5406 Å), and the diffraction pattern was recorded over a 2θ range of 10–80°. The surface morphology of the HCCDs was examined using scanning electron microscopy (SEM) (JeoL, Tokyo, Japan). Energy-dispersive X-ray (EDX) spectroscopy, in conjunction with SEM, was used to determine elemental composition and elemental maps, confirming the distribution of carbon, nitrogen, and oxygen. In addition, particle size and morphology were evaluated using transmission electron microscopy (TEM) by placing a drop of the particle suspension on a carbon-coated copper grid, allowing it to freeze, then drying it for imaging.

#### 2.2.2. Antibacterial Activity Against Oral Pathogen *S. mutans*

The antimicrobial efficacy of the synthesized HCCDs was evaluated against clinical isolates of *S. mutans* obtained from the Department of Microbiology at Saveetha Medical College and Hospital, using the agar well diffusion method. The overnight cultures of the test pathogens were diluted to 0.5 McFarland turbidity standard (~1 × 10^8^ CFU/mL) and spread uniformly on Mueller–Hinton agar (MHA) plates. The 6 mm diameter wells were located aseptically, and the carbon dot suspensions were placed inside the wells at different concentrations (20–80 μg/mL). Sterile distilled water was used as a negative control, and standard antibiotics (e.g., streptomycin, 30 μg) were used as a positive control. The plates were then incubated at 37 °C for 24 h, after which the zones of inhibition (ZOI) were measured in mm [21].

#### 2.2.3. Minimum Inhibitory Concentration (MIC) Assay

The minimum inhibitory concentration (MIC) of HCCDs against *Streptococcus mutans* was determined using the broth microdilution method in sterile 96-well microtiter plates according to the Clinical and Laboratory Standards Institute (CLSI) guidelines, with minor modifications. Briefly, HCCDs were prepared at an initial concentration of 1000 μg/mL and subjected to two-fold serial dilutions in sterile brain heart infusion (BHI) broth to obtain final concentrations ranging from 1000 to 1.7 μg/mL (1000, 500, 250, 125, 62.5, 31.25, 15.62, 7.81, 3.90, and 1.7 μg/mL). An overnight culture of *S. mutans* was adjusted to approximately 1 × 10^6^ CFU/mL, and 100 μL of the standardized bacterial suspension was added to each well containing 100 μL of the respective HCCD dilution. Wells containing a bacterial suspension without HCCDs served as the negative control, and the positive control was defined as *S. mutans* with streptomycin added. The plates were incubated at 37 °C for 24 h under appropriate conditions. Following incubation, bacterial growth was assessed by adding 20 μL of 0.015% (*w*/*v*) resazurin solution to each well and incubating for an additional 20 min. A change in colour from blue to pink indicated bacterial growth, whereas wells retaining the blue colour indicated inhibition of bacterial growth. The MIC was defined as the lowest concentration of HCCDs that completely prevented visible bacterial growth, as evidenced by the absence of resazurin colour change [22].

#### 2.2.4. Antibiofilm Activity

The culture medium was carefully aspirated after incubation, and the tooth was rinsed with 1× PBS to remove any cells not adherent to the tooth. The biofilms were then stained with a SYTO9 and propidium iodide (PI) staining solution and incubated for 15 min in the dark, at room temperature. The *S. mutans* were then washed with 1× PBS, and this was repeated a second time. In the end, the stained biofilms were examined using confocal laser scanning microscopy (CLSM).

#### 2.2.5. Cell Line

The human osteosarcoma (MG-63 cell line) was purchased from NCCS, Pune, India. The cells were grown in Dulbecco’s Modified Eagle Medium (DMEM) supplemented with 10% fetal bovine serum (FBS) and 1% penicillin–streptomycin. The cultures were incubated in a humidified CO_2_ incubator at 37 °C and 5% CO_2_.

#### 2.2.6. Cytotoxicity Assessment by MTT Assay

To assess the cytotoxic effect of the HCCDs, approximately 5000 MG-63 human osteosarcoma cells/well were plated in 96-well plates and allowed to grow until a confluency of about 70%. Cells were exposed to the different concentrations of HCCDs and incubated for 24 h in a CO_2_ incubator. After treatment, 10 μL of MTT solution (5 μg/mL) was added to each well, and the plate was incubated in the dark for 2 h. A total of 100 μL of DMSO was then added to dissolve the purple formazan crystals, and the medium was then carefully removed. A microplate reader was used to measure absorbance at 570 nm, and a percentage was calculated to determine cell viability.

#### 2.2.7. Morphological Observation

For morphological analysis, 1 × 10^6^ cells (MG-63) were cultured in 6-well plates and allowed to reach 70% confluency. The MG-63 cells were then incubated with the HCCDs for 24 h. After treatment, cells were observed under an inverted light microscope (Euromex, Arnhem, The Netherlands) at 20× magnification.

#### 2.2.8. Live/Dead Assay

MG-63 cells were treated with HCCDs and stained with a solution containing Acridine Orange (AO) and PI (4 μL) to detect Cell death. The cells were then left in the dark for more than 20 min, after which they were gently washed with 1× PBS to remove excess dye. The stained cells were then analysed with a Confocal Laser Scanning Microscope (Automated Di8, Leica, Grove IL, USA).

#### 2.2.9. Wound-Healing Assay

In 6-well plates, cells (MG-63) were grown at a density of 1 × 10^5^ cells/well to a confluency of about 95%. To duplicate a wound, a uniform scratch was then created in the middle of the well using sterile 200 μL pipette tips. The culture liquid was aspirated carefully, followed by a careful rinse with phosphate-buffered saline (PBS), before the unattached cells were removed from the wells. Then, 1.5 mL of serum-free medium with or without HCCDs was added to each well as a control. Images of the wound area were taken at the first time point (t_0_) and 24 h later (t_24_) using a phase-contrast microscope equipped with a 10× magnification.

#### 2.2.10. Statistical Analysis

The data have been presented as the mean ± standard deviation from three independent experiments. Statistical significance was analysed in GraphPad Prism version 8.4.2 using one-way ANOVA, followed by Tukey’s test to determine *p <* 0.05.

## 3. Result and Discussion

### 3.1. UV-Visible and Fluorescence Property of HCCDs

**Figure 1:**
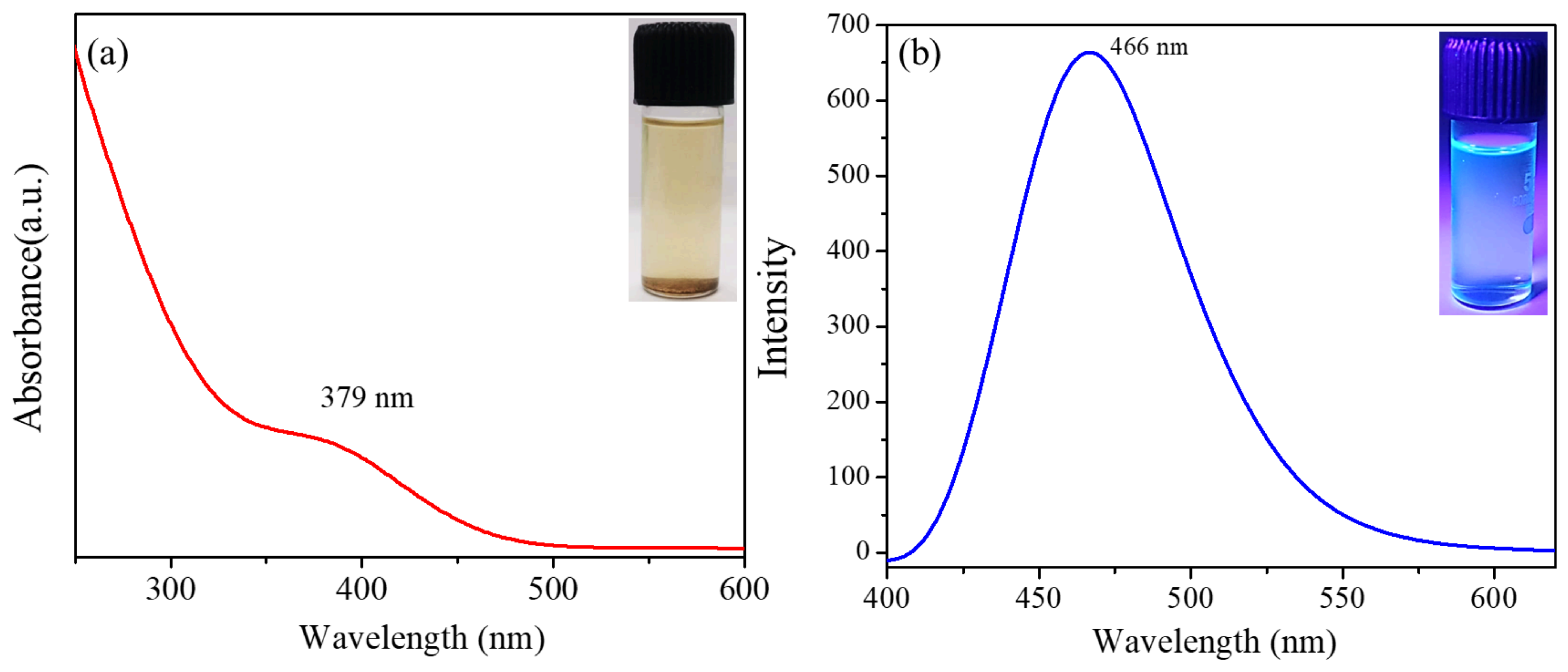
(**a**) UV–Vis and (**b**) photoluminescence spectra of HCCDs.

The HCCDs exhibited excellent optical properties, enabling high-level fluorescence-based bioimaging owing to their strong excitation-dependent PL. UV–visible spectroscopy showed a characteristic absorption band around 379 nm which is indicative of n→π* electronic transitions of surface states that have carbonyl groups (Figure 1a). The particles exhibited a significant fluorescence peak at ~466 nm upon excitation at 380 nm, yielding strong blue emission, as shown in Figure 1b. This is due to the different emissive traps and defect states in the surface created during nanoscale carbonization processes, which exhibit heterogeneous excitation-dependent fluorescence behaviors.

### 3.2. Surface Functional and Crystalline Properties of HCCDs

**Figure 2:**
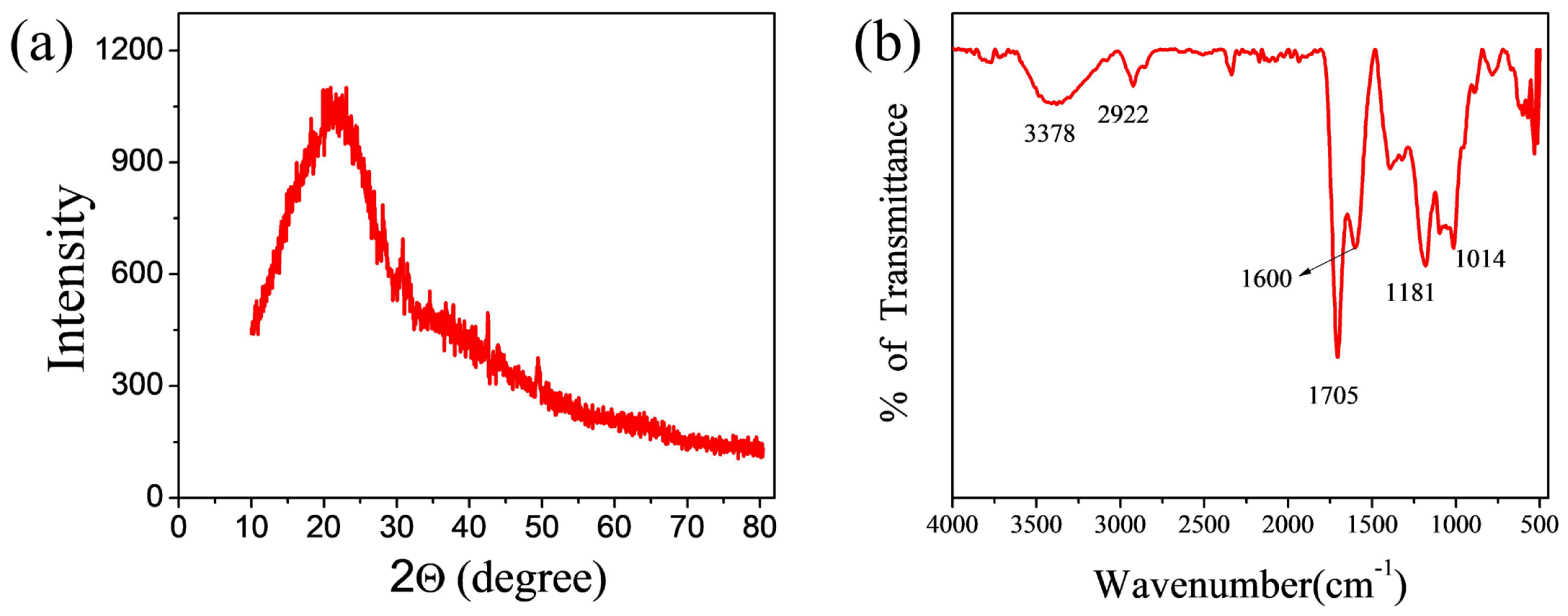
(**a**) XRD analysis. (**b**) FTIR analysis of HCCDs.

The X-ray diffraction (XRD) pattern exhibited a broad diffraction peak without any distinct sharp reflections, indicating the predominantly amorphous nature of the synthesized CDs (Figure 2a). A broad peak at 2θ ≈ 23.449° was observed, attributed to the (002) plane of graphitic carbon. The disordered sp^2^-hybridised carbon domains with short-range graphitic stacking are indicated by this diffraction characteristic.

FTIR spectrum of the synthesized carbon dots showed that the various functional groups were present on the surface (Figure 2b). A broad absorption band at 3378 cm^−1^ is attributed to O–H and N–H stretching vibrations, suggesting the presence of hydroxyl and amine functionalities on the surface of the CDs. The C–H stretching vibration of aliphatic groups is associated with the peak at 2922 cm^−1^. The strong peak at 1705 cm^−1^ is assigned to the carbonyl or carboxylic acid groups C=O stretching vibration, and the peak at 1600 cm^−1^ is attributed to C=C stretching vibration of the sp^2^ carbon domain in aromatic structure. Moreover, the peaks at 1181 and 1014 cm^−1^ are attributed to the stretching vibrations of C–O and C–N bonds, respectively, further indicating the successful surface functionalization of the carbon dots with epoxy and oxygen-containing groups.

### 3.3. Structural Property of HCCDs

**Figure 3:**
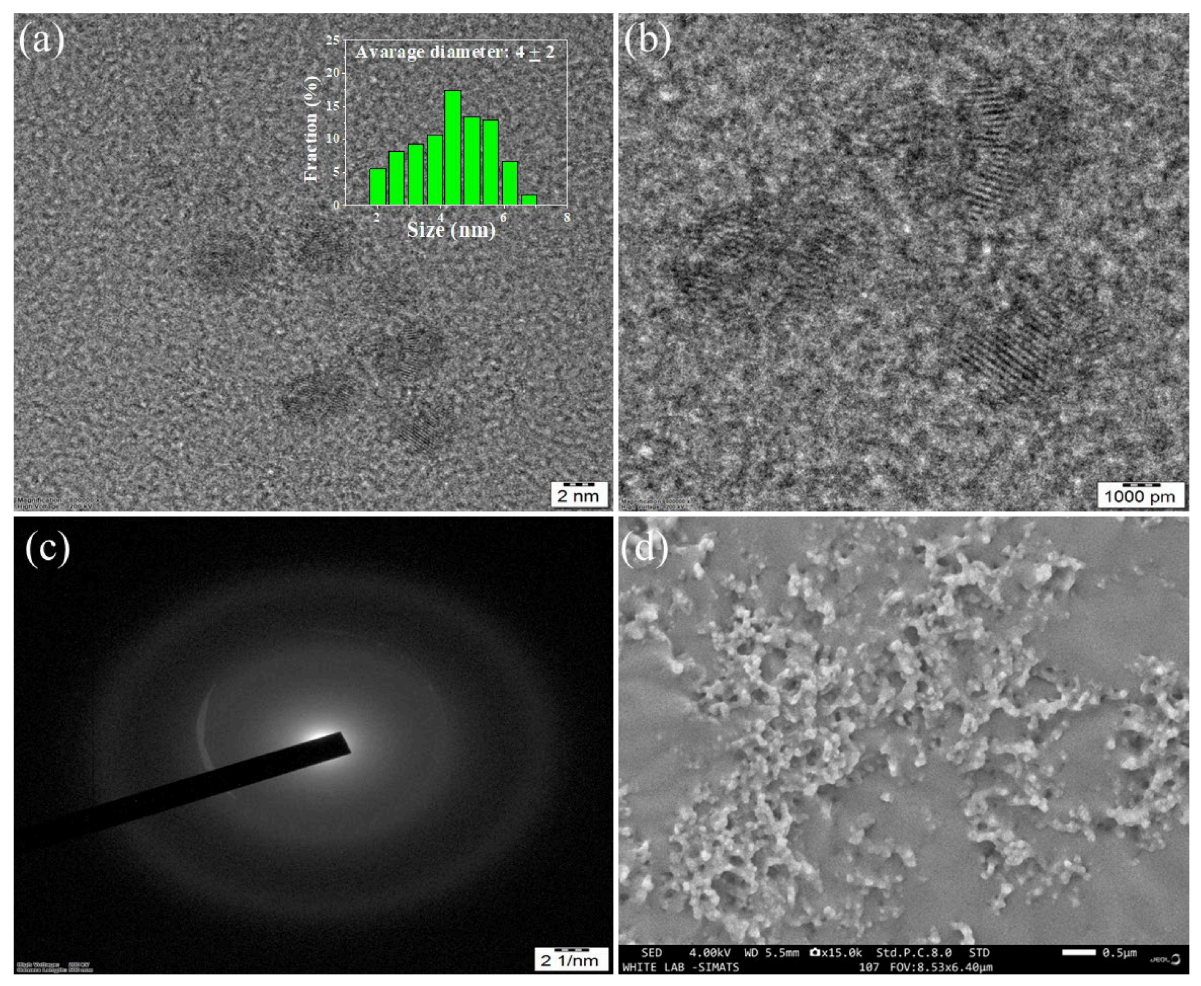
(**a**,**b**) HRTEM, (**c**) SAED, and (**d**) SEM analyses reveal crystalline, uniformly distributed HCCDs with nanoscale structural ordering.

Morphology and microstructural characteristics of the synthesized carbon dots were studied by TEM, HRTEM, SAED and SEM analysis (Figure 3). The TEM image (Figure 3a) showed that the carbon dots were nearly spherical, evenly distributed, and slightly aggregated. The average particle diameter from the particle size histogram was about 4 ± 2 nm, which further confirmed the nanosized nature of the synthesized CDs. In the high-resolution TEM (HRTEM) image shown in Figure 3b, the lattice fringes became more visible, indicating the partially crystalline nature of the carbon dots. The observed interlayer spacing is attributed to the presence of graphitic carbon domains, indicating that an ordered carbon structure with sp2-hybridised carbon atoms forms within the amorphous carbon matrix. A selected area electron diffraction (SAED) pattern was acquired (Figure 3c), with a few diffused concentric rings indicating the presence of a large amount of amorphous carbon and some degree of graphitic crystallinity. It was also found that carbon dots are agglomerated, likely due to their reduced surface energy and nanosized structure, as shown in the SEM image (Figure 3d). The particles were well distributed and exhibited a compact granular structure, demonstrating the successful synthesis of nanoscale carbon materials.

### 3.4. EDAX and Mapping of HCCDs

**Figure 4:**
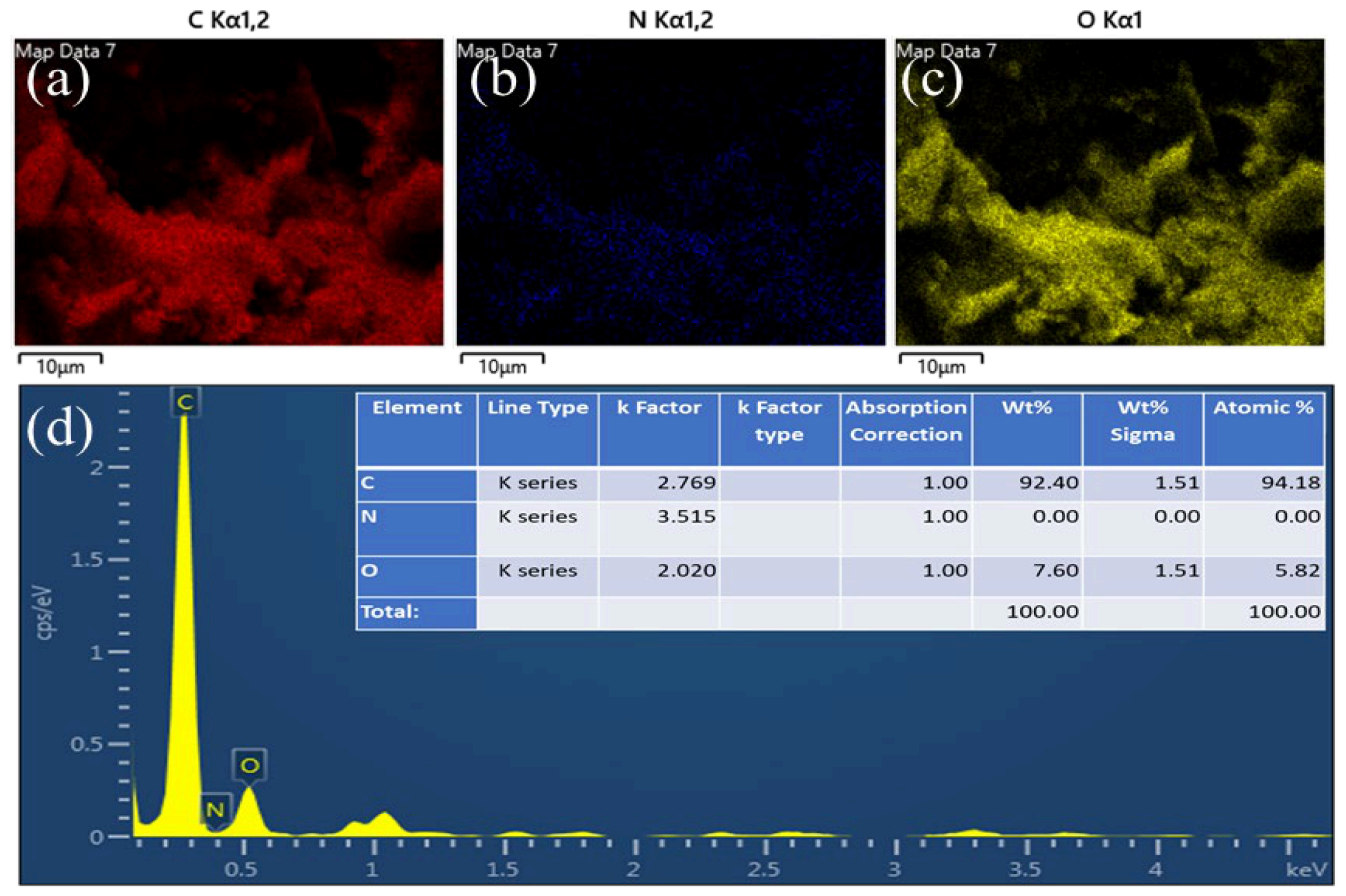
EDX spectra and elemental mapping of HCCDs, (**a**) Carbon (Red), (**b**) nitrogen (Blue), (**c**) Oxygen (Yellow), and (**d**) EDAX of carbon dots, and percentage of elemental composition of HCCDs.

Elemental composition and distribution of the synthesized carbon dots were determined by energy-dispersive X-ray spectroscopy (EDX/EDAX) and elemental mapping (Figure 4). The elemental mapping images showed a uniform distribution of carbon and oxygen across the sample. Carbon mapping of the synthesized nanomaterial is consistent with the main composition of carbon, and another large and uniform carbon signal is observed (Figure 4a). There is nearly no nitrogen signal from the nitrogen mapping (Figure 4b), which indicates that there is little or no N in the carbon dots. In the mapping of oxygen in Figure 4c, the surface of the CD is evenly covered with oxygen-containing functional groups. The elemental quantification results from the EDAX also confirmed the elemental composition of the carbon dots, as seen in their spectrum (Figure 4d). A very prominent carbon peak was detected, along with a smaller oxygen peak. The result of quantitative analysis showed that the synthesized CDs had a composition of 92.40 wt% carbon and 7.60 wt% oxygen with atomic percentages of 94.18% and 5.82%, respectively. The high carbon content further indicates successful formation of carbon nanostructures, and the oxygen content is consistent with the FTIR results, which showed oxygen-containing surface functional groups.

### 3.5. X-Ray Photoelectron Microscopy

**Figure 5:**
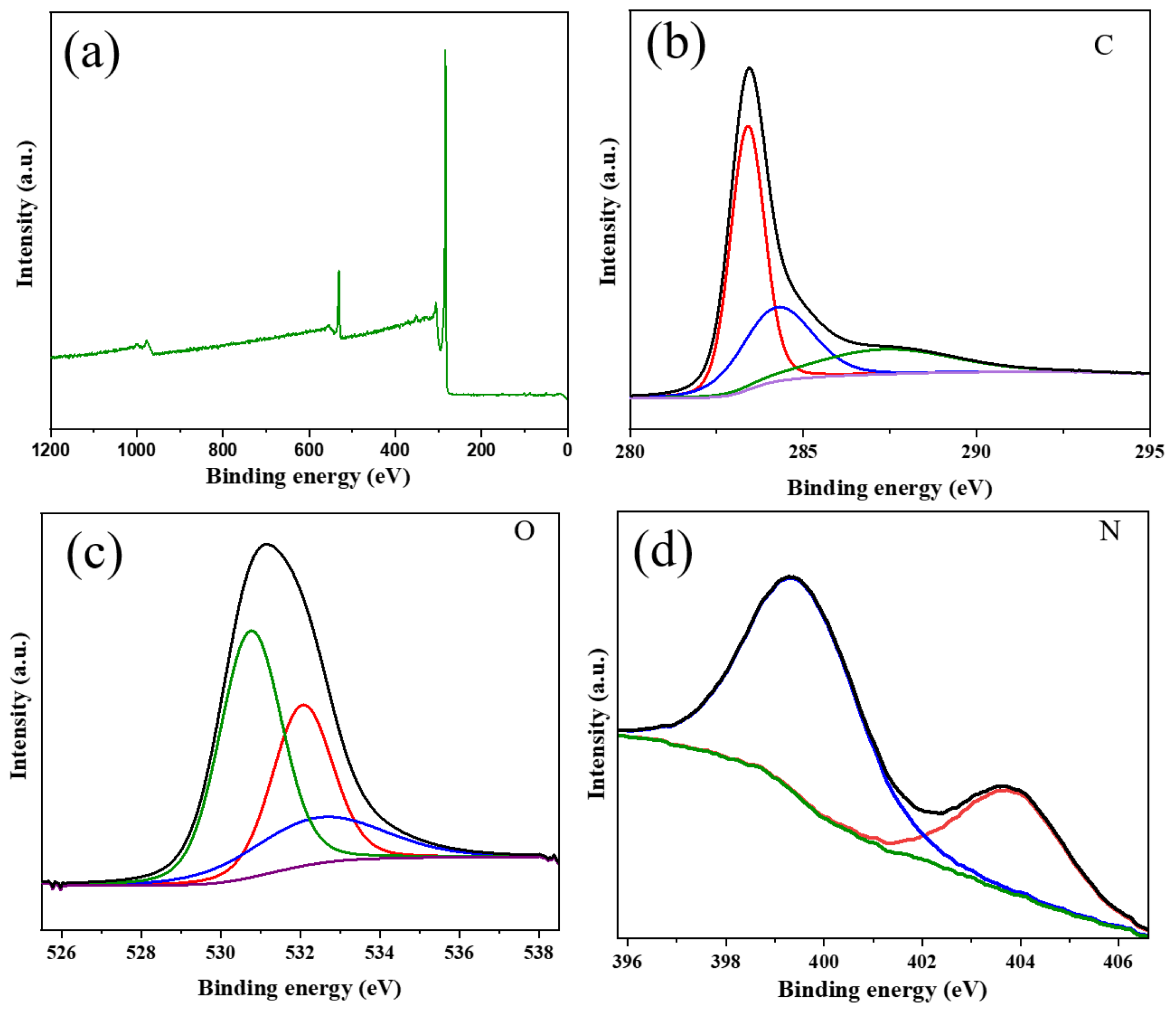
XPS characterization of HCCDs: (**a**) survey spectrum and deconvoluted high-resolution spectra of (**b**) C 1s, (**c**) O 1s, and (**d**) N 1s, confirming the surface elemental composition and bonding states.

The surface chemical composition and electronic states of the synthesized HCCDs were investigated by X-ray photoelectron spectroscopy (XPS). The survey spectrum in Figure 5a clearly shows that the HCCDs are composed of C, N and O elements, and the measured amounts of each element (89.17 at. % C, 0.84 at. % N and 10.99 at. % O) demonstrate that the nitrogen is incorporated successfully, while a high proportion of oxygen-containing surface functionalities is present. The synthesized carbon dots were found to be highly pure, as seen in the absence of extra impurity peaks. The coexistence of nitrogen and oxygen heteroatoms in the carbon framework is expected to regulate the electronic structure, enhance the density of surface-active sites and their hydrophilicity, thereby benefiting their fluorescence behavior and biological performance. A high-resolution C 1s spectrum (Figure 5b) contained three contributions at 283.38, 284.33 and 287.28 eV. The central peak located at 283.38 eV is attributed to sp^2^ hybridized graphitic carbon (C=C), indicating the formation of a conjugated carbon core. The main peak at 284.33 eV is attributed to C-C/C-N covalent bonds, suggesting the successful incorporation of N into the carbon lattice. The other peak at a higher binding energy, that is, at 287.28 eV, is assigned to carbonyl (C=O) species, indicating that the HCCD surface contains more oxygen-rich functional groups.

These surface functionalities make the particles readily dispersible in water, provide a large number of reactive sites, and promote further interactions with bioactive molecules. The deconvoluted N 1s spectrum (Figure 5c) shows two well resolved peaks located at 399.3 and 403.8 eV. The lower BE peak at 399.3 eV is attributed to pyrrolic/pyridinic nitrogen, introducing structural defects in the graphitic structure and introducing additional chemically active sites. The second peak at 403.8 eV is assigned to oxidized nitrogen (N–O), which is formed during the hydrothermal synthesis process. Nitrogen incorporation has been shown to effectively modify the electronic distribution of the carbon network, improve charge-transfer characteristics and create defect-rich active centres which are responsible for the excellent optical characteristics and increased surface reactivity of the HCCDs. The high-resolution O 1s spectrum (Figure 5d) can be deconvoluted into three peaks: one at 530.78 eV, another at 532.08 eV, and the third at 532.60 eV. The carbonyl oxygen (C=O) is responsible for the peak at 530.78 eV, while the peaks at 532.08 and 532.60 eV are attributed to C–O–C/C–OH and hydroxyl or adsorbed oxygen species at the surface, respectively. The high-density oxygen-based functional groups significantly increase the hydrophilicity and colloidal stability of the HCCDs and provide numerous sites for hydrogen bonding and surface functionalization.

The presence of these oxygen functionalities is also presumed to facilitate interactions with the bacterial cell membrane and reactive oxygen species, thereby enhancing the antibacterial, antibiofilm, and antioxidant activities of the HCCDs. In sum, the XPS results clearly help to validate the successful synthesis of nitrogen-doped, oxygen-rich HCCDs that feature a partially graphitized carbon structure decorated with copious oxygen-containing functional groups. The graphitic carbon, defect sites created by nitrogen and oxygenated surface functionalities create a synergistic effect that increases the electronic properties, surface polarity and chemical reactivity of the HCCDs. These structural arrays enable their outstanding fluorescence properties and high stability in aqueous media, and they are multifunctional for biomedical applications such as antibacterial agents, antibiofilm agents, antioxidants, and bioimaging.

### 3.6. Antimicrobial Activity of HCCDs

**Figure 6:**
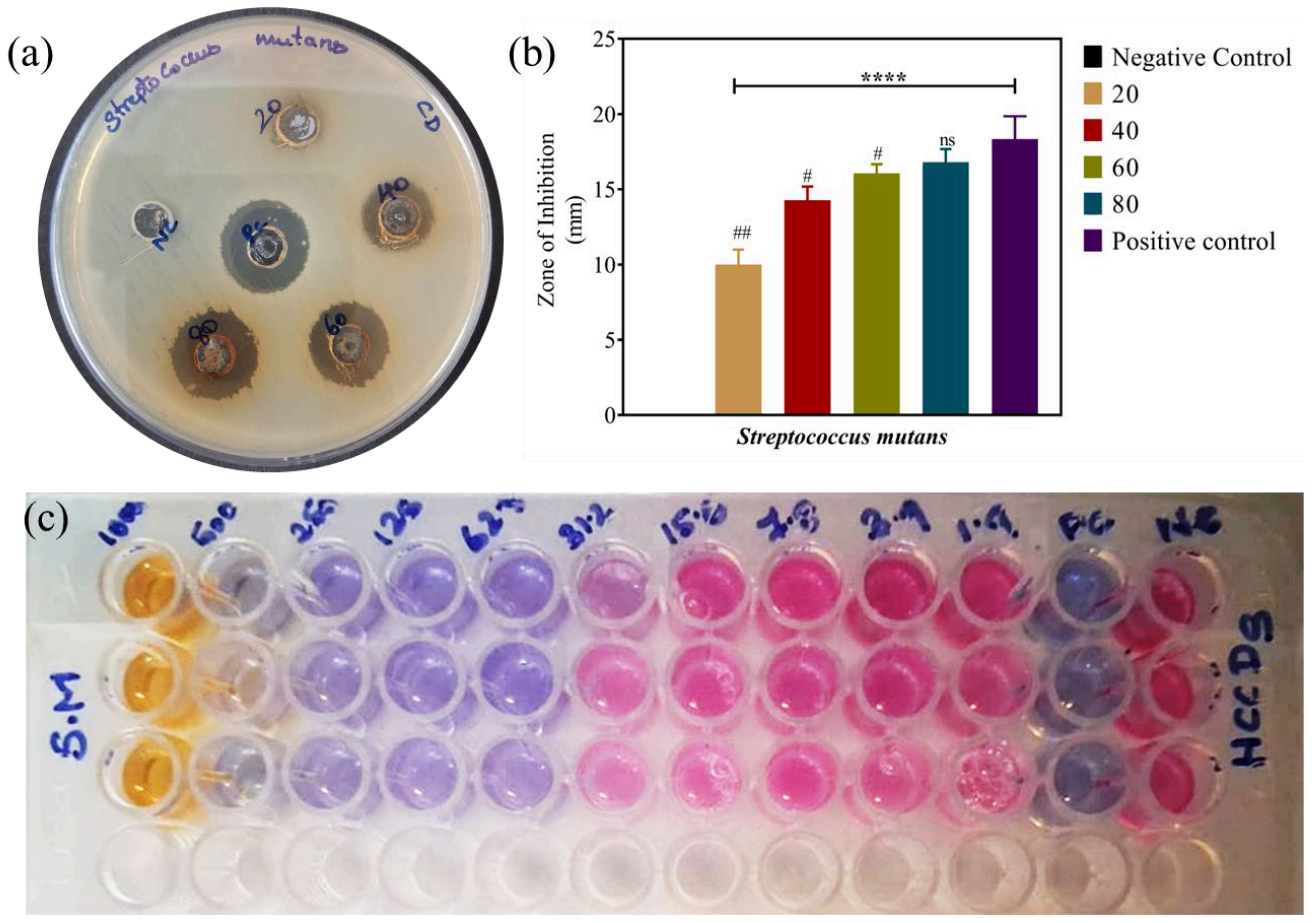
(**a**) Antimicrobial activity of Synthesized HCCDs using agar well diffusion. (**b**) The graph represents the zone of inhibition, mean ± Standard deviation, of three independent experiments. The statistical significance **** denotes *p* ≤ 0.0001 negative control vs. treated groups, ns denotes non -significance, # denotes *p* ≤ 0.05, and ## denotes *p* ≤ 0.01 between Positive control vs. treated groups. (**c**) HCCDs have a significant antibacterial activity against cariogenic bacteria and can inhibit the growth of bacteria at low doses.

The antibacterial activity of the HCCDs against *S. mutans* was evaluated using the agar well diffusion technique, and the results showed concentration-dependent inhibition of *S. mutans* growth (Figure 6a). The zones of inhibition at 20, 40, 60, and 80 µg/mL were 10.0 ± 0.81 mm, 14.2 ± 0.75 mm, 16.0 ± 0.49 mm, and 16.8 ± 0.71 mm, respectively, while the positive control showed a zone of 18.3 ± 1.4 mm. A negative control group showed no inhibition zone (Figure 6b), demonstrating the good antibacterial activity of HCCDs against the test bacteria.

The antibacterial activity of the HCCDs against *S. mutans* was evaluated using the broth microdilution method, with MICs determined by the absence of growth in the main culture, as indicated in Figure 6c. The MIC value for HCCDs was defined as the lowest concentration that showed neither colour change nor bacterial turbidity. The results showed that the growth of *S. mutans* could be inhibited in a concentration-dependent manner by HCCDs. HCCDs demonstrated a strong antibacterial effect against cariogenic bacteria at high concentrations, at which no bacterial growth was observed due to concentrations exceeding the MIC. The tested concentration that completely prevented growth was 62.5 µg/mL against *S. mutans*.

### 3.7. Antibiofilm Activity of HCCDs

**Figure 7:**
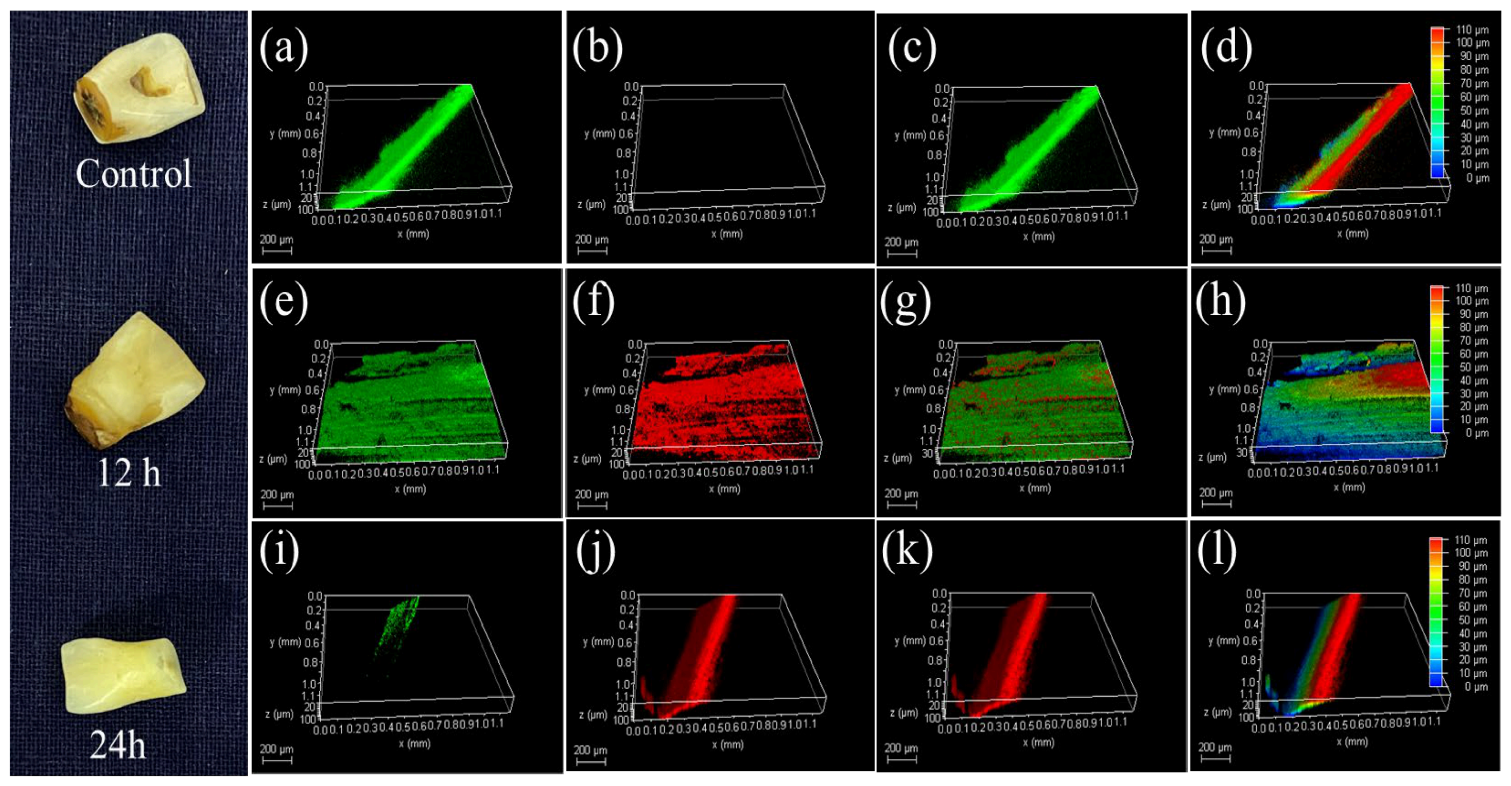
The CLSM analysis of *S. mutans* biofilms on tooth surfaces after treatment with HCCDs at 100 µg mL^−1^ at different time intervals shows time-dependent antibacterial and antibiofilm activity. Dense, viable biofilms with substantial thickness are seen in the control groups (**a**–**d**). Partial disruption of the biofilm, with cell inactivation, is observed after 12 h (**e**–**h**), followed by an increase in PI-positive dead cells. (**i**–**l**) At 24 h, extensive membrane-compromised bacterial death, pronounced biofilm thinning, and near-complete structural collapse confirm potent HCCDs-mediated biofilm eradication. Scale bar @ 200 μm.

Figure 7a–l presents the CLSM analysis of *S. mutans* biofilm treated with HCCDs using SYTO 9/PI dual staining. SYTO 9 stains viable bacterial cells with green fluorescence, whereas propidium iodide (PI) penetrates membrane-compromised cells and emits red fluorescence, indicating dead bacteria. The untreated control group were mainly seen with intense green fluorescence with the dense and well-organized biofilm architecture, which confirmed the presence of viable bacterial colonies and the formation of a mature biofilm.

Bacterial cell death was negligible, as minimal red fluorescence was observed in the control. Following 12 h of HCCDs treatment, a significant decrease in green fluorescence intensity along with an increase in red fluorescence intensity were detected. The overall structure of the biofilm had been disorganised and disrupted, indicating that HCCDs were effective at altering the integrity of the bacterial cell membrane and inhibiting biofilm maturation. The combined CLSM images also revealed the presence of both live and dead bacterial cells, suggesting that the antibacterial activity of the carbon dots gradually increased over time. The biofilm at 24 h of treatment showed a predominant red signal with only a few green signals, indicating a high level of bacterial death and severe disruption of the biofilm matrix. The three-dimensional reconstruction images also showed a marked decrease in biofilm thickness and surface coverage compared with the control group. These results indicate that HCCDs exhibit potent antibiofilm activity and a bactericidal effect against *S. mutans*, inhibiting bacterial viability in a time-dependent manner and preventing the maturation of *S. mutans* biofilm.

### 3.8. Cell Viability Assay for Carbon Dots in MG-63 Cells

**Figure 8:**
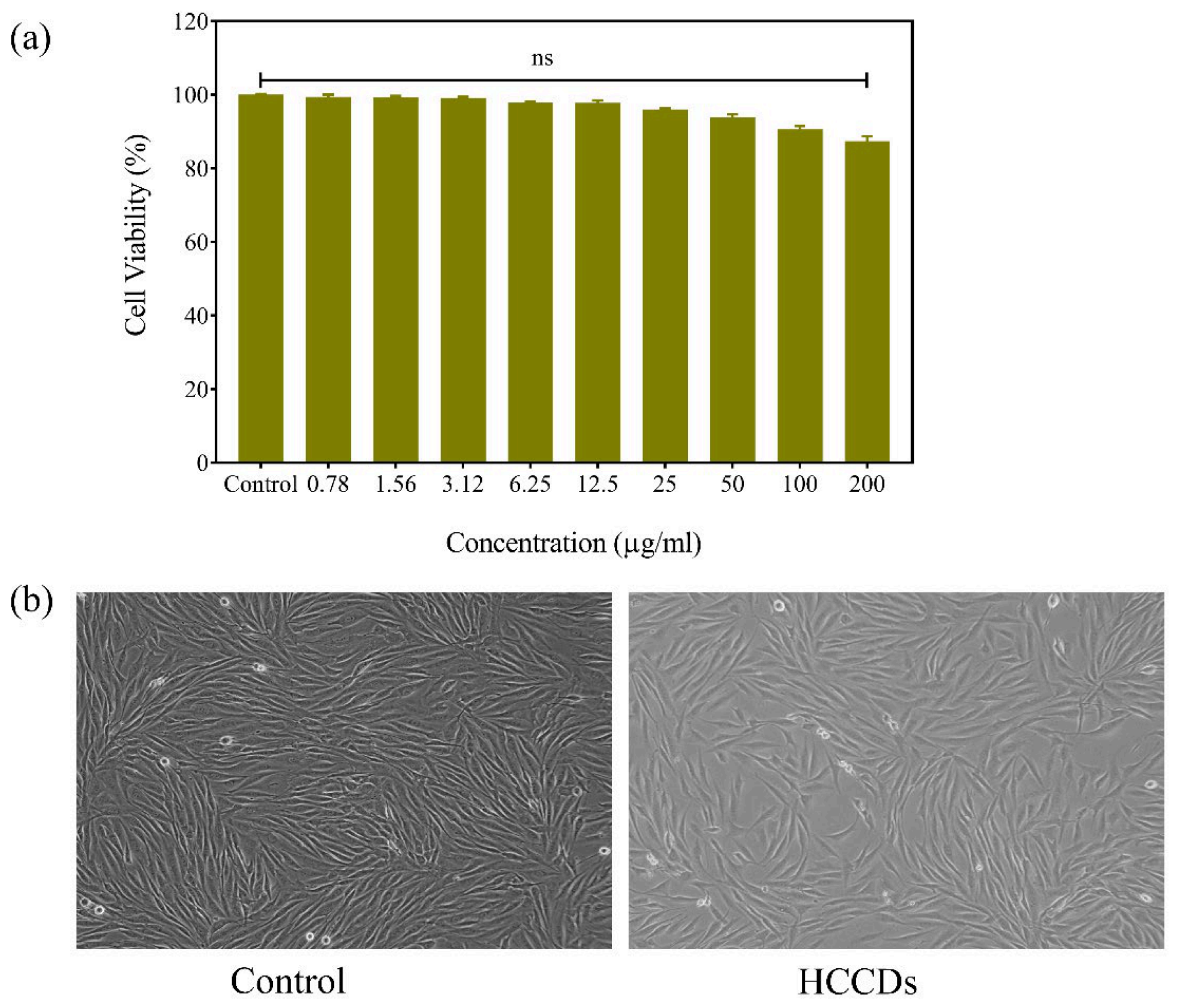
(**a**) Cytotoxicity effect of HCCDs in the MG-63 cell line by MTT assay. The data were represented as the mean ± standard deviation from a triplicate experiment. No significant change was observed in the control and HCCD-treated groups. (**b**) Morphological changes in HCCDs. Scale bar at 20× magnification.

To evaluate the cytocompatibility of the synthesized HCCDs, a cell viability assay was performed on MG-63 cells (Figure 8a). The results showed that the carbon dots were not cytotoxic towards MG-63 cells up to 200 µg/mL. The synthesized HCCDs showed good biocompatibility, with cell viability exceeding 86% even at 200 µg/mL. The morphology of control and carbon dot-treated MG-63 cells were observed, as shown in Figure 8b. The cells in both groups showed normal morphology, were well spread and adherent, and showed no shrinkage or evidence of damage or detachment following treatment. The results further corroborate the excellent biocompatibility and cellular safety of the synthesized carbon dots.

### 3.9. Live and Death Assay in MG-63 Cells

**Figure 9:**
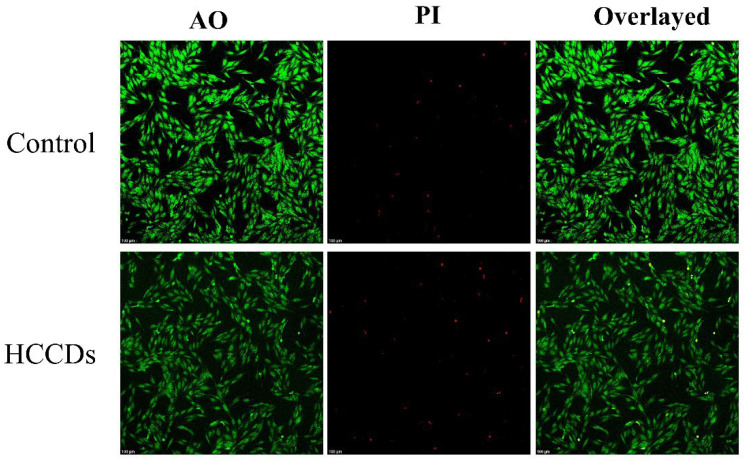
Dual staining (AO/PI) for live and dead assay in the MG-63 cell line with and without HCCDs. Scale bar at 100 µm.

The synthesized carbon dots HCCDs were also tested for their cytocompatibility towards MG-63 cells by acridine orange/propidium iodide (AO/PI) live–dead staining assay (Figure 9). In the control group, they observed very strong green fluorescence, indicating that the cell membranes were intact and that the cells were viable and healthy. Likewise, the fluorescence was mainly green in the HCCD-treated MG-63 cells. When assessing significant differences in the number of red- or orange-fluorescent cells, no significant difference was found between the treated and control groups. The results show that the HCCDs carbon dots exhibit good biocompatibility and low cytotoxicity toward MG-63 cells.

### 3.10. Wound Healing Assay

**Figure 10:**
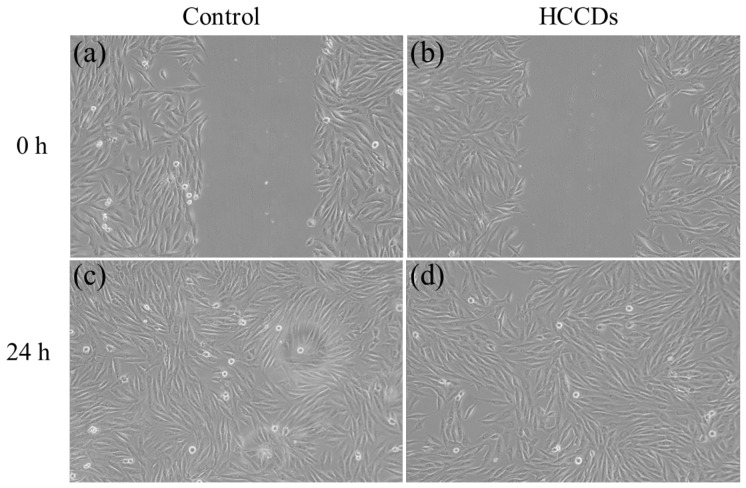
In vitro scratch (wound healing) assay demonstrating migration of MG-63 cells under (**a**,**c**) control, and (**b**,**d**) HCCDs-treated group, showing accelerated wound closure after 24 h without compromising normal cell morphology. Scale bar at 20× magnification.

The ability of the synthesized HCCDs to heal wounds was tested in MG-63 cells using an in vitro scratch assay (Figure 10). A clear scratch area was seen in both groups (control and HCCD-treated) at 0 h. The control group exhibited a remarkable wound closure after 24 h of incubation, during which the migratory ability of MG-63 cells was observed. At the same time, wound closure was similar in the HCCDs-treated group, suggesting that HCCDs treatment did not affect cell migration or proliferation. The good cytocompatibility and non-toxicity of the synthesized HCCDs were proven by the fact that the cultured cells showed normal cell behavior and migrated efficiently toward the scratched region. The results indicate that HCCDs are biocompatible and could be used in biomedical and tissue engineering applications.

## 4. Discussion

Natural precursors, hyaluronic acid and clove extract, were used in the present study during the green synthesis of HCCDs. The excellent biocompatibility, hydrophilicity and oxygen-containing functional groups of HA contributed to the water dispersibility and cellular compatibility of the synthesized carbon dots. As a eugenol-, flavonoid-, and phenolic-abundant extract, clove served as a natural carbon source and facilitated surface functionalization. The combination of these biomaterials enabled the creation of biocompatible, functionalized carbon nanostructures for biomedical applications. It has been shown that plant extracts are effective precursors for the synthesis of sustainable carbon dots because they contain natural aromatic and polyphenolic compounds that can serve as graphitic carbon nuclei during thermal treatment [23]. There have been previous attempts to prepare biocompatible fluorescent carbon dots using tea leaves extract [24], turmeric [25], orange peel [26], and clove, with multifunctional properties such as Metal sensing, Antimicrobial activity, and Photodegradation of dye.

The XRD analysis of the synthesized HCCDs showed a broad diffraction peak at around 23°, suggesting a relatively high degree of amorphous nature of the carbon material with some graphitic ordering. These wide diffraction patterns are typically seen in carbon dots. The same has been reported for carbon dots prepared via biomass-derived materials, with the disordered sp^2^ carbon domains leading to broad (002) graphitic reflections [27]. This was confirmed by HRTEM analysis, which showed lattice fringes of graphitic carbon domains within an amorphous carbon matrix. We observed the average particle size to be about 4.17 nm when analyzed using TEM, indicating successful synthesis of HCCDs, which is in agreement with previously reported green synthesis of carbon dots [28]. The nanoscale size is beneficial for biological applications, as smaller particles increase cellular uptake and fluorescence and are less cytotoxic [29].

FTIR analysis revealed that there were a large number of hydroxyls, carbonyl, carboxyl, and C–O functional groups on the surface of the HCCDs. These functional groups containing oxygen are probably the result of the decomposition and carbonization of hyaluronic acid and phytochemicals in clove extract. The optical properties, stability in water and biological activity of carbon dots are strongly influenced by the presence of surface functional groups. The N-rich surface functionalities displayed by carbon dots obtained from plant extract have previously been reported to give rise to good water solubility and biocompatibility properties [30]. Moreover, these functional groups can promote hydrogen-bonding interaction with biological membrane and extracellular matrix components which can improve the bio compatibility of the cells.

The EDAX and elemental mapping analysis results showed that the major element in synthesized HCCDs was carbon, followed by oxygen as the secondary element. Uniform elemental distribution suggests homogeneous carbonization as well as successful formation of oxygen-functionalized carbon nanostructures. Elemental compositions similar to those of the carbon dots obtained in this work have been reported for the carbon dots derived from biomass using green hydrothermal methods [31].

The biocompatibility testing was performed in MG-63 osteoblast-like cells, showing that the synthesized HCCDs were non-toxic even at higher concentrations, up to 200 µg/mL, with cell viability higher than 86%. No significant cytotoxic effects were observed, suggesting that HA and the clove extract produced highly biocompatible carbon dots for biological applications. Low toxicity was also reported in previous studies of natural carbon dots, with concentrations up to 500 μg/mL in the Hacat cell line [32]. The results of the AO/PI live–dead staining assay also supported this, as the majority of the treated cells were green, with very few yellow- or orange-stained cells, indicating high cell survival. The appearance of the treated cells after preservation also confirms the cytocompatibility of the synthesized HCCDs, because a normal cellular morphology and cell adherence are clearly observed. Previously, Chitosan and eugenol-based carbon dots showed a similar low cytotoxicity effect in the 3T3 Cell line [33]. Interestingly, the wound-healing assay revealed that MG-63 cells treated with HCCD showed wound closure nearly identical to that of the untreated control group within 24 h. The data indicate that the synthesized carbon dots do not affect cellular migration and proliferation. There are also reports that carbon dots extracted from plant stalks can promote wound healing due to their anti-inflammatory and antioxidant properties [34]. Another study has shown that carbon dots can mediate M2 macrophage polarization to enhance wound healing and possess antibacterial activity [35].

The results showed the multifunctional properties of the synthesized HCCD, suggesting their great potential as an endodontic nanomaterial. Persistence of bacterial biofilms in the root canal space, especially those of cariogenic and endodontic pathogens, remains a major hurdle to successful treatment of a root canal treatment. Hyaluronic acid and phytochemicals extracted from civet are a combination of a potent antibacterial activity, antibiofilm activity, and excellent cytocompatibility, in a synergistic combination. Moreover, the oxygen-rich surface functional groups and nanoscale size of HCCDs can enable them to penetrate into intricate dentinal tubules and/or biofilm matrices, which indicates possible applications in intracanal disinfection, as an additive to root canal irritants, as well as a regenerative endodontic biomaterial. This finding holds promise for the translational relevance of HCCDs in improving infection control while maintaining biocompatibility for endodontic tissue regeneration.

## 5. Conclusions

To conclude, the synthesized HCCDs demonstrated their outstanding multifunctional activity with potent antimicrobial and anti-biofilm activity, along with cytocompatibility. HCCDs showed an inhibitory effect on the growth of *S. mutans* (Minimal Inhibitory Concentration (MIC) of 62.5 µg/mL) and no harmful effect on the viability of MG-63 cells or on normal cell migration, whereas the biofilm architecture was effectively altered after incubation in the presence of the optimum concentration of HCCDs. The combined action of hyaluronic acid and bioactive compounds from cloves results in a biocompatible nanoplatform with potential for translation into endodontic disinfection, dental biofilm control in root canal therapy, and regenerative dental treatment. This discovery paves the way for HCCDs to play a major role in future nanotherapeutics in endodontia, and additional preclinical and in vivo studies will still be needed before they can be applied clinically.

## Data Availability

The original contributions presented in this study are included in the article. Further inquiries can be directed to the corresponding author.
